# Extracellular Vesicles of *Porphyromonas gingivalis* Disrupt Trophoblast Cell Interaction with Vascular and Immune Cells in an In Vitro Model of Early Placentation

**DOI:** 10.3390/life13101971

**Published:** 2023-09-27

**Authors:** Brenda Lara, Matías Sassot, Guillermina Calo, Daniel Paparini, Laura Gliosca, Gabriela Chaufan, Iñaki Loureiro, Daiana Vota, Rosanna Ramhorst, Claudia Pérez Leirós, Vanesa Hauk

**Affiliations:** 1 Universidad de Buenos Aires—CONICET, Instituto de Química Biológica de la Facultad de Ciencias Exactas y Naturales (IQUIBICEN), Buenos Aires C1428EGA, Argentina; brendalara1308@gmail.com (B.L.); matiassassot@gmail.com (M.S.); guillecalo@yahoo.com.ar (G.C.); daniel.paparini@gmail.com (D.P.); laura.gliosca@odontologia.uba.ar (L.G.); rgchaufan@gmail.com (G.C.); inakiloureiro@gmail.com (I.L.); daianavota@gmail.com (D.V.); rramhorst@hotmail.com (R.R.); 2 Universidad de Buenos Aires, Facultad de Odontología, Cátedra de Microbiología, Buenos Aires C1122AAH, Argentina

**Keywords:** *P. gingivalis* outer membrane vesicles, placentation, trophoblast cells, neutrophils

## Abstract

Extracellular vesicles released by the primary pathogen of periodontal disease *Porphyromonas gingivalis* (*Pg*), referred to as outer membrane vesicles (OMVs), have been associated with the pathogenesis of systemic diseases like cardiovascular disease, rheumatoid arthritis, and Alzheimer’s disease. A pathogenic role for *Pg* by disrupting placental homeostasis was proposed in the association between periodontal disease and adverse pregnancy outcomes. On the basis that trophoblast-derived factors modulate endothelial and immune cell profiles in normal pregnancy and the scarce presence of *Pg* in placenta, we hypothesized that OMVs from *Pg* affect trophoblast cell phenotype, impairing trophoblast–endothelium and trophoblast–neutrophil interactions. By means of in vitro designs with first-trimester human trophoblast cells, endothelial cells, and freshly isolated neutrophils, we showed that *Pg* OMVs are internalized by trophoblast cells and modulate the activity and expression of functional markers. Trophoblast cells primed with *Pg* OMVs enhanced neutrophil chemoattraction and lost their anti-inflammatory effect. In addition, reduced migration with enhanced adhesion of monocytes was found in endothelial cells upon incubation with the media from trophoblast cells pretreated with *Pg* OMVs. Taken together, the results support a pathogenic role of *Pg* OMVs at early stages of pregnancy and placentation through disruption of trophoblast contribution to vascular transformation and immune homeostasis maintenance.

## 1. Introduction

Extracellular vesicles released by pathogenic and commensal bacteria act as mediators for the delivery of molecular signals among bacteria or between bacteria and the host, establishing an efficient cell-free intercellular communication [1,2,3]. *Porphyromonas gingivalis* (*Pg*), a Gram-negative facultative anaerobe of the oral cavity, is considered a primary pathogen of periodontal disease [4,5]. *Pg* produces extracellular vesicles, most commonly referred to as outer membrane vesicles (OMVs), which are released into the environment where it grows and spread to distant sites, suggesting a pathogenic role in the pathogenesis of systemic diseases [6]. OMVs from *Pg* (*Pg* OMVs) have been implicated in diabetes, cardiovascular disease, rheumatoid arthritis and Alzheimer’s disease [6,7,8,9].

Accumulating evidence and meta-analysis support an association between periodontal disease and adverse pregnancy outcome, including preeclampsia and preterm birth, with high risk of maternal and neonatal mortality [10,11,12]. In particular, a pathogenic role for *Pg* was reported from studies on human pregnancy and mouse pregnancy models [13,14,15].

Homeostasis maintenance during placentation relies on the cooperative interaction of maternal leukocytes recruited to the decidua, vascular cells, and the trophoblast [16,17]. Likewise, trophoblast cells differentiate to an invasive phenotype that interacts with vascular endothelial cells as a crucial step for blood vessel transformation and adequate placentation [18,19]. On the other hand, trophoblast-derived local factors modulate the monocyte profile [17,20] as well as neutrophil recruitment and their functional shaping to anti-inflammatory and tissue-repairing phenotypes to maintain placental homeostasis [21,22]. In line with this, inflammation caused by bacteria or viruses is associated with trophoblast failure to migrate and invade the decidua and to acquire an endothelium-like phenotype that contributes to blood vessel remodeling. This failure underlies the development of pregnancy complications like preeclampsia, fetal growth restriction, and preterm birth, among other related disorders [16,23].

Although the presence of bacterial extracellular vesicles in human term placenta of uncomplicated pregnancies has recently been reported [24], there is no reference to the presence or to the effect of OMVs from *P. gingivalis*. Therefore, the aim of this work was to study the effect of *Pg* OMVs on trophoblast–endothelium and trophoblast–immune interactions by means of in vitro designs with first-trimester human trophoblast cells, human endothelial cells, and freshly isolated human neutrophils.

The evidence presented here supports a pathogenic role of *Pg* OMVs at early stages of pregnancy and placentation through disruption of trophoblast contribution to vascular transformation, on the one hand, and on the other, impairing their immunomodulatory effect on neutrophil activation to maintain immune homeostasis.

## 2. Materials and Methods

### 2.1. Porphyromonas gingivalis Outer Membrane Vesicle Isolation

*P. gingivalis* strain ATCC 33277 was cultured in anaerobic conditions at 37 °C using brain–heart infusion (BHI; Oxoid) medium supplemented with 5% l-cysteine, 5 mg/L hemin, and 1 mg/L menadione until it reached the early stationary phase (OD_600nm_). The culture samples were then centrifuged at 8000× *g* for 10 min at 4 °C to eliminate bacteria, and the supernatants containing *Pg* OMV were filtered through a 0.22 μm filter (GE Healthcare Life Sciences, Boston, MA, USA). Subsequently, ultracentrifugation was performed for 70 min at 100,000× *g* at 4 °C. Once the pellet was resuspended in PBS, the *Pg* OMVs were sterilized using a 0.22 μm filter and then frozen at −80 °C. The protein concentration of *Pg* OMVs was quantified using the Pierce bicinchoninic acid (BCA) protein assay kit. DNA and RNA were quantified via UV absorbance using a NanoDrop 2000/2000c (Thermo Fisher Scientific, Waltham, MA, USA). Quantifications were carried out using samples from three independent cultures of *P. gingivalis*. Transmission electron microscopy was used to visualize *Pg* OMVs. Briefly, the samples were adsorpted on a carbon-coated copper grid that was washed with PBS and negatively stained with uranyl acetate (*w*/*v* 1%). Particle size distribution was obtained by dynamic light scattering (DLS) analysis using Malvern Zetasizer ZS90 (Malvern Panalytical Ltd., Malvern, UK).

### 2.2. Trophoblast, THP-1 Monocyte, and Endothelial Cell Cultures

The HTR-8 trophoblast cell line was cultured in Dulbeco’s modified Eagle’s medium and Nutrient Mixture F-12 (DMEM-F12) (Life Technologies, Grand Islands, NY, USA) supplemented with 10% heat-inactivated fetal bovine serum and 100 U/mL streptomycin-100 µg/mL penicillin solution, the endothelial cell line EA.hy926 was cultured in Iscove’s Modified Dulbecco’s Medium (IMDM) with 10% FBS in the presence of 100 U/mL penicillin G and 100 mg/mL streptomycin, and the human monocytic cell line THP-1 (ATCC TIB-202) was cultured in RPMI 1640 medium supplemented with 10% heat-inactivated FBS (*v*/*v*), 100 U/mL penicillin, 100 μg/mL streptomycin, 1% glutamine, 250 mg/mL glucose, 11 mg/L sodium pyruvate, and 50 μM β-mercaptoethanol. All cell cultures were maintained at 37 °C and 5% CO_2_. The HTR-8 cell line was kindly provided by Dr. Gil Mor (Wayne State University, Detroit, MI, USA) and the Ea.y926 cell line was supplied by Dr. Fernanda Parborell (IBYME-CONICET, Buenos Aires, Argentina).

### 2.3. Confocal Microscopy

*Pg* OMVs were labeled with the lipid intercalating dye PKH67 according to the manufacturer’s instructions. Cells were incubated with the pre-stained vesicles for 8 h at 37 °C. Then, the cells were washed, fixed with paraformaldehyde 4%, and stained with tetramethylrhodamine isothiocyanate–phalloidin and DAPI. The cells were visualized via confocal fluorescence microscopy.

### 2.4. Trophoblast-Conditioned Media Preparation

To obtain trophoblast-conditioned media (Tb CM), 4 × 10^5^ cells were pretreated for 6 h in the presence/absence of 1 μg/mL *Pg* OMVs. Then, the cells were exhaustively washed 5 times with PBS and grown for another 24 h in RPMI 2% FBS to generate Tb CM for trophoblast–neutrophil interaction studies or in DMEM 0% FBS for trophoblast–endothelial interaction studies. CM were collected and stored at −80 °C.

### 2.5. Neutrophil Isolation

Neutrophils were isolated from healthy donors by Ficoll-Paque PLUS (GE Healthcare, IL, USA) gradient centrifugation and Dextran (MP Biomedicals, Göteborg, Sweden) sedimentation, followed by hypotonic lysis, as previously described [22,25]. Neutrophils were suspended in RPMI 1640 medium supplemented with 100 U/mL penicillin, 100 μg/mL streptomycin, and 10% heat-inactivated FBS. The Argentine Society of Clinical Investigation Board and Ethical Committee approved the study. All healthy donors provided written informed consent.

### 2.6. Neutrophil Migration Assay

Neutrophil migration was quantified using a transwell migration assay. Briefly, 2 × 10^5^ neutrophils were seeded in transwells with 3 μm pores that were placed in a lower chamber containing no treatment (control), Tb CM, or *Pg* OMV-pretreated Tb CM (*Pg* OMV CM). After 30 min at 37 °C, the number of migrated neutrophils was determined via flow cytometry.

### 2.7. Reactive Oxygen Species (ROS) Production

Neutrophils (2 × 10^5^) were incubated at 37 °C with Tb CM or *Pg* OMV Tb CM for 30 min. Then, 5 μM 2′,7′-dichlorofluorescin diacetate (DCFH-DA) (Sigma-Aldrich, St. Louis, MA, USA) was added for another 15 min, and then the cells were washed and immediately analyzed via flow cytometry [21].

### 2.8. qPCR

Total RNA was extracted from the different cell types using TriReagent. RNA was retrotranscribed to obtain cDNA. For qPCR assays, cDNAs were incubated with Bio-Rad SYBR Green Master Mix and specific primers. The threshold cycle method (2^−ΔΔCt^ method) was used to determine the relative gene expression levels. Table 1 depicts the sequence of the primers used [22,25,26].

### 2.9. Antioxidant Enzyme Activity

For the determination of the enzyme activity of the catalase, cells were cultured to confluence in the presence or absence of *Pg* OMVs for 24 h. Subsequently, the cells were lysed, followed by centrifugation at 11,000× *g*, and the assays were conducted on the resulting supernatants. Catalase (CAT, EC1.11.1.6) activity was assessed by monitoring the decomposition of hydrogen peroxide at 240 nm. The reaction mixture comprised 50 mM potassium phosphate buffer (pH 7.0) and 30 mM hydrogen peroxide. The outcomes were quantified as UCATs (catalase units) per mg of protein [27,28]. UCATs were defined as the amount of enzyme degrading 1 mmol of peroxide per minute.

### 2.10. MTT Assay

Cell viability was determined via the conversion of 3-(4,5-dimethylthiazol-2-yl)-2,5-diphenyl tetrazolium bromide (MTT) dye. Following the stimulation period, MTT was added to the wells, reaching a final concentration of 1 mg/mL, and subsequently incubated at 37 °C for 2 h. The reaction product was solubilized using dimethyl sulfoxide, and the optical density was measured at 570 nm.

### 2.11. Endothelial Cell Migration

A wound-healing assay was performed to study endothelial cell migration. Briefly, EA.hy926 cells were grown to confluence in 24-well plates and then a wound was made in the monolayer with a sterile tip. Cells were washed twice with PBS and incubated with DMEM 0% FBS (control), Tb-CM, or *Pg* OMV Tb-CM. Microphotographs were taken at 0 and 18 h post scratching, the images were analyzed using ImageJ^®^ software (ImageJ 1.53c Wayne Rasband, National Institutes of Health, Bethesda, MD, USA, http://imagej.nih.gov/ij), and the wound closure was calculated as described previously [25].

### 2.12. Monocyte Adhesion Assay

Endothelial Ea.hy926 cells were stimulated for 24 h in the presence of Tb CM, in the presence of *Pg OMV* Tb CM, or with no treatment (control). Then, THP-1 cells (approximately 10^5^ cells) were labeled with calcein AM (5 μM in PBS) for 15 min at 37 °C. Subsequently, the cells were washed twice with RPMI medium and resuspended in 100 µL of medium before being added to EA.hy926 cultures for 1 h. Then, the endothelial cells were carefully washed with pre-warmed PBS to remove non-adherent THP1. In total, 100 µL of PBS were added to each well and microphotographs were taken with a fluorescent microscope. Adherent monocytes were counted, and the results are shown as the fold change with respect to the untreated cells.

### 2.13. Statistical Analysis

The data were analyzed using GraphPad Prism 9 software (GraphPad, San Diego, CA, USA). The paired *t*-test or one-way ANOVA with Sidak’s multiple comparisons was used for parametric analysis. Wilcoxon or ANOVA with Dunn´s comparisons was used for non-parametric assays. Results are expressed as mean ± S.E.M. Differences between treatments were considered significant at *p* < 0.05.

## 3. Results

### 3.1. Porphyromonas gingivalis Outer Membrane Vesicle Isolation and Internalization by Trophoblast Cells

**Figure 1:**
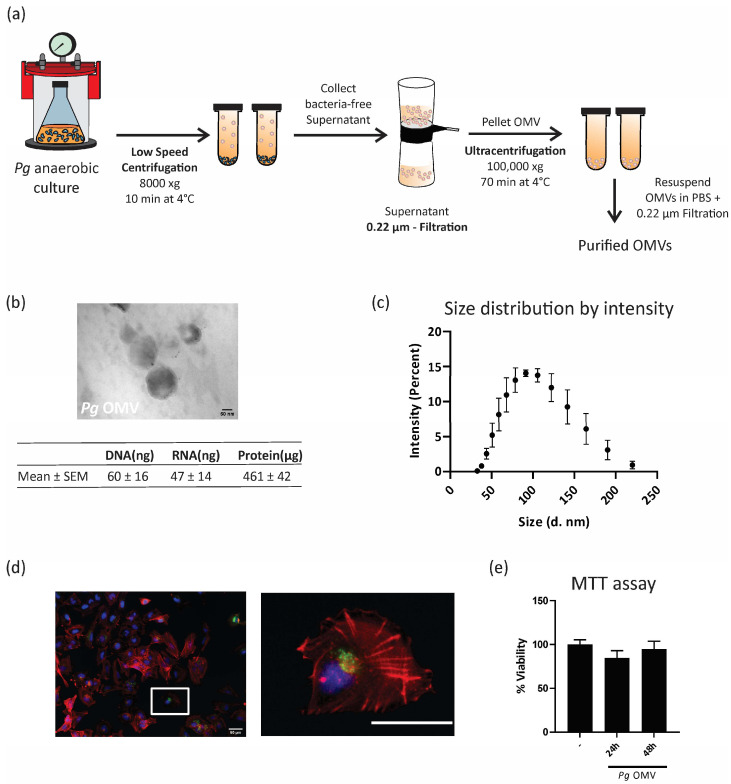
***Porphyromonas gingivalis* outer membrane vesicle isolation and characterization.** (**a**) *Pg* OMVs were isolated according to the protocol described in Section 2. (**b**) A representative image of the transmission electron microscopy is shown. Scale bar = 50 nm. Magnification: 250,000×. Lower table: total DNA, RNA, and protein obtained from 500 mL of three different bacterial cultures. (**c**) Analysis of the size distribution of *Pg* OMVs via DLS (*n* = 3) (diameter in nm). (**d**) Confocal microscopy of HTR-8 treated with PKH67-labelled (green) Pg-OMVs for 9 h. Cells were stained with phalloidin to visualize the actin cytoskeleton (red) and DAPI for the nuclei (blue). Left image: Lower magnification. Scale bar: 50 μm. Right image: Higher magnification image of the area marked with the with rectangule. Scale bar: 50 μm. (**e**) Cell viability in the control (–) or after 24/48 h of *Pg* OMV treatment (1 μg/mL) was assessed by MTT assay. % viability was determined by taking the control (–) average absorbance value as 100%.

*Porphyromonas gingivalis* outer membrane vesicles (*Pg* OMVs) were isolated from bacteria anaerobic culture supernatants using an ultracentrifugation protocol, as illustrated in Figure 1a. Purified *Pg* OMVs were visualized using transmission electron microscopy, which indicated nanoscale spheric structures (Figure 1b). As depicted in the table of Figure 1b, the *Pg* OMV cargo included biologically active products such as proteins, DNA, and RNA, which are key molecules for intercellular communication. Figure 1c shows the size distribution range assessed by DLS, from 35 to 220 nm in diameter, with an average size of 108 ± 39 nm. Our next question was whether trophoblast cells internalize *Pg* OMVs. Figure 1d shows confocal microscopy images of first-trimester human trophoblast cells (HTR-8/SVneo cell line) stained with phalloidin to visualize the actin cytoskeleton (red) and DAPI for the nuclei (blue). As can be seen in Figure 1d, trophoblast cells incorporated *Pg* OMVs (1 μg/mL) stained with PKH67 (green) at 8 h, and they remained internalized up to 24 h (not shown). A detail of the internalized *Pg* OMV nanostructures is shown in the right image. As expected, incubations with *Pg* OMVs at concentrations of 0.1 μg/mL and 10 μg/mL resulted in fewer and larger incorporations of vesicles, as assessed in confocal images. Based on this, 1 μg/mL was selected for the next functional experiments as the lowest concentration, which was rapidly internalized (6–9 h) and visible in confocal images. Furthermore, the viability of the trophoblast cells was not affected at 24 or 48 h of incubation (Figure 1e).

### 3.2. Pg OMVs Modulate Trophoblast Cell Oxidative State and Unbalance Trophoblast Marker Expression

**Figure 2:**
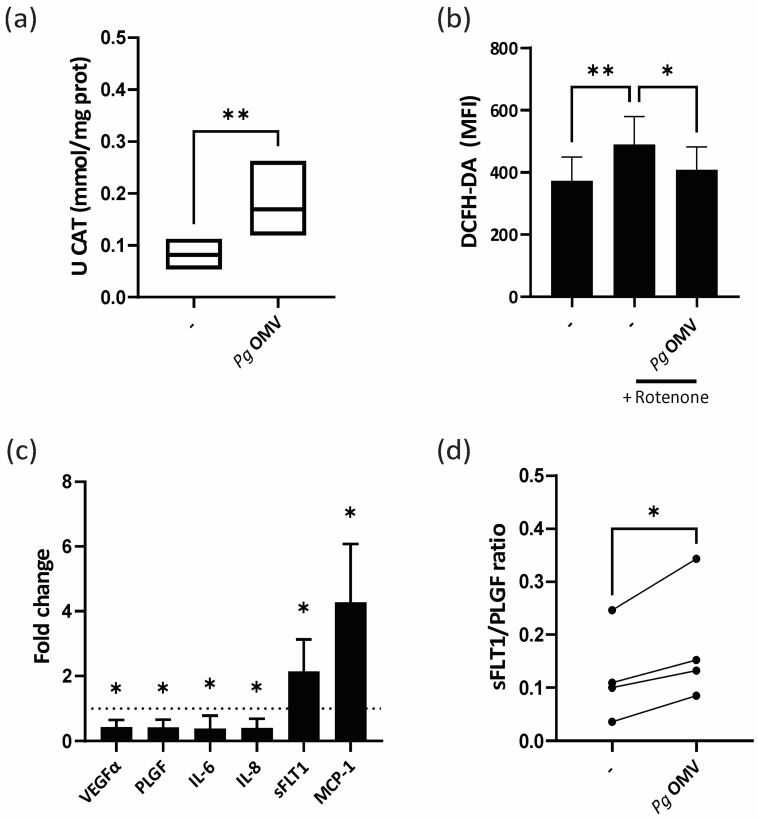
***Pg* OMVs modulate trophoblast oxidative state and promote trophoblast marker expression imbalance**. (**a**) HTR-8 cells were treated with 1 µg/mL *Pg* OMVs for 24 h in DMEM-F12 2% FBS. Antioxidant enzyme activity was measured as described in Section 2 (Materials and Methods). *n* = 6; ** *p* < 0.01; paired *t*-test. (**b**) Trophoblast cells were preincubated for 24 h *Pg* OMVs. Then, rotenone (200 uM) was added for 1 h and total ROS levels were measured via flow cytometry using DCFH-DA. (**c**) HTR-8 trophoblast cells were incubated for 6 h in the presence/absence of 1 μg/mL *Pg* OMVs. mRNA levels of VEGF-a, PLGF, IL-6, IL-8, sFLT-1, and MCP-1 were assessed via qPCR. Values shown represent fold change of cells treated with *Pg* OMV relative to cells in absence of *Pg* OMV (dotted line). *n* = 4, *t*-test. (**d**) sFLT1/PLGF ratio calculated for basal (−) or *Pg* OMV-treated HTR-8 trophoblast cells. * *p* < 0.05; ** *p* < 0.01; paired *t*-test.

Cumulative evidence indicates that anaerobic bacteria, particularly Pg, modulate antioxidant enzymes and scavenging molecules of host cells as a survival strategy [29,30]. On this basis, we next assessed the potential of *Pg* OMVs to suppress or prevent the formation of reactive oxygen species in trophoblast cells, focusing on the activity of the key enzymes catalase (CAT) and superoxide dismutase (SOD) and the levels of the scavenging molecule reduced glutathione (GSH). Figure 2a shows that incubation of trophoblast cells for 24 h with 1 μg/mL *Pg* OMVs increased the activity of catalase. There was no effect of *Pg* OMVs on GSH levels, whereas superoxide dismutase activity showed a trend of reduced activity. To dive deeper into the oxidative stress modulation by *Pg* OMVs, rescue experiments were conducted by pre-treating trophoblast cells with *Pg* OMVs for 24 h before exposing them to the oxidative stress inducer rotenone. As expected, rotenone alone increased total ROS levels in the trophoblast cells (Figure 2b). However, this increase was significantly attenuated when the trophoblast cells were pre-treated with *Pg* OMVs, suggesting a protective antioxidant or oxygen scavenger effect of *Pg* vesicles (Figure 2b).

Given the modulatory effect of *Pg* OMV on oxidative balance in trophoblast cells, we next wondered whether the synthesis of mediators such as cytokines, chemokines, and angiogenic factors, which are known to correlate with oxidative levels [31], were also altered. We measured the expression of cytokines IL-8 and IL-6, the neutrophil and monocyte chemoattractant cytokine MCP-1, the proangiogenic factor VEGF-a, and the placental-related factors soluble fms-like tyrosine kinase 1 (sFlt-1) and placental growth factor (PlGF) via qPCR. Figure 2c shows that *Pg* OMV treatment induced a selective modulation of placental markers, with reduced expression of IL-8, IL-6, PlGF, and VEGF-a accompanied by increased expression of s-Flt-1 and MCP-1 (Figure 2c). On the basis that the sFlt-1:PlGF ratio has been approved as a diagnostic aid in conjunction with other clinical findings for preeclampsia and validated as a predictive test [32], the sFlt-1:PlGF ratio was calculated and it was found to be increased in four independent assays (Figure 2d).

### 3.3. Trophoblast Cells Primed with Pg OMVs Enhance Neutrophil Chemoattraction and Activation

**Figure 3:**
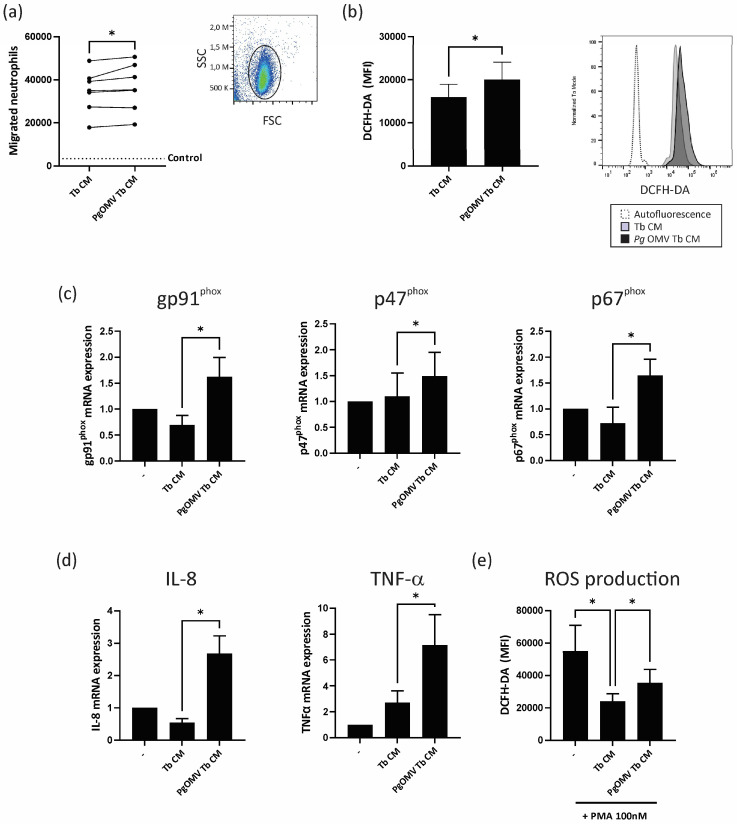
***Pg* OMV favors neutrophil migration and activation.** (**a**) Neutrophils were allowed to migrate towards Tb-conditioned media for 30 min through a 3 μm transwell and cells of the lower compartment were counted via flow cytometry. The gating strategy for the neutrophil population is shown. *n* = 7; * *p* < 0.05; Wilcoxon test. (**b**) Neutrophils were stimulated for 45 min with Tb CM or *Pg* OMV Tb CM, and ROS production was quantified via flow cytometry using 2′,7′-dichlorofluorescin diacetate, as indicated in Materials and Methods. Representative histograms are shown. Values are mean ± S.E.M. *n* = 9; * *p* < 0.05; Wilcoxon test. (**c**,**d**) Neutrophils were incubated for 6 h with Tb CM or *Pg* OMV Tb or were untreated (–), and mRNA expression of gp91^phox^, p47^phox^, p61^phox^ (**c**), Il-8, and TNF-α (**d**) was evaluated by qPCR. Values are mean ± S.E.M. *n* = 6; * *p* < 0.05; Friedman test. (**e**) Neutrophils were pretreated for 15 min with Tb CM or *Pg* OMV Tb CM or were untreated (–) and then were stimulated for 45 min with PMA 100 ng/mL. ROS production was assessed using DCFH-DA as previously described. Values are mean ± S.E.M. *n* = 9; * *p* < 0.05; Friedman test.

Considering that activated neutrophils have a detrimental impact on trophoblast cell function [22,33,34], our next question was whether soluble factors secreted by trophoblast cells conditioned by *Pg* OMVs promote neutrophil chemoattraction and activation. We used an experimental design using conditioned media from trophoblast cells preincubated with *Pg* OMVs and freshly isolated circulating neutrophils. As shown in Figure 3a, the conditioned media from trophoblast cells pretreated with *Pg* OMVs (*Pg* OMV Tb CM) increased neutrophil chemoattraction compared with control trophoblast-conditioned media (Tb CM). To determine whether *Pg* OMV Tb CM also induced neutrophil activation, we evaluated ROS production and cytokine/chemokine expression in neutrophils. *Pg* OMV Tb CM induced higher ROS production (Figure 3b) and increased mRNA expression of gp91^phox^ p47^phox^, and p67^phox^, subunits of the NADPH oxidase complex (Figure 3c). The activation of this complex generates the majority of ROS production in neutrophils [35]. In addition, *Pg* OMV Tb CM augmented IL-8 and TNF-α expression, two neutrophil activation markers (Figure 3d). Furthermore, in previous studies, we demonstrated that trophoblast-conditioned media deactivates PMA-activated neutrophils by inhibiting ROS release and NET formation [21]. Here, we observed that trophoblast-conditioned media from *Pg* OMV-pretreated cells lost the capacity to deactivate neutrophils, as an increase in ROS production after PMA stimulation was observed in *Pg* OMV Tb CM-treated neutrophils compared to those treated with control Tb CM (Figure 3e). The absence of the remaining *Pg* OMVs in trophoblast cell-conditioned media was checked via fluorimetry.

### 3.4. Trophoblast Cells Primed with Pg OMVs Impair Endothelial Cell Migration and Activation

**Figure 4:**
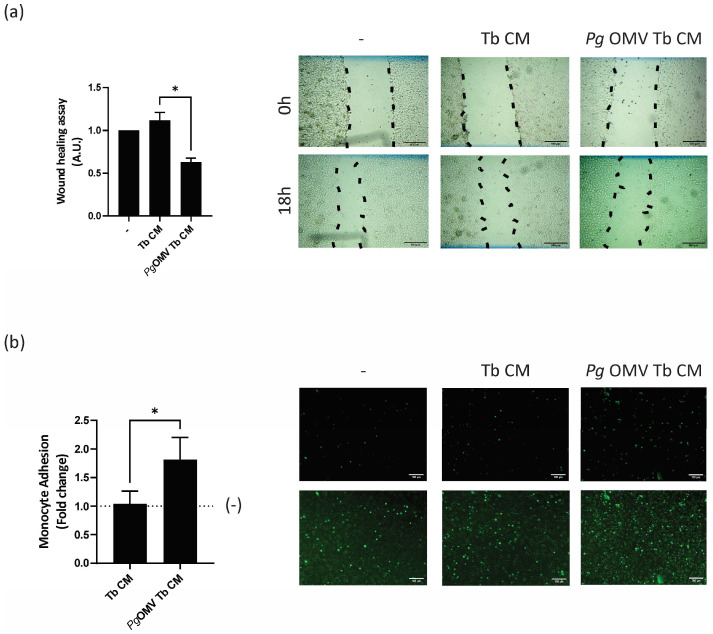
**Deleterious effect of *Pg* OMVs on trophoblast–endothelial crosstalk.** (**a**) A wound-healing assay was performed to evaluate endothelial cell migration. Ea.hy926 cells were grown until confluence. Then, the cells were wounded and incubated for 0–18 h in DMEM 0%FBS (–) or in the presence of Tb CM or *Pg* OMV Tb CM. Representative images from the assay at 0 h and after 18 h of scratching. Black lines represent the migration fronts. * *p* < 0.05; *n* = 4. (**b**) The EA.hy926 cells were pre-treated with Tb CM or *Pg* OMV Tb CM for 24 h. The control group (–) was untreated cells. THP-1 cells were labeled with green calcein AM (5 μM) and co-cultured with endothelial cells for 1 h to allow adhesion. Representative pictures from green calcein AM-labeled THP-1 cells adhered to EA.hy926 cells under fluorescence microscope (upper) or microscope (lower) * *p*  <  0.05; *n* = 4; scale bar  =  100 μm.

Spiral artery remodeling at the maternal–fetal interface is crucial for the success of a pregnancy and depends on the interaction between first-trimester trophoblast cells and maternal vessel endothelial cells [16]. Given the imbalance in the expression of pro- and anti-angiogenic mediators induced by *Pg* OMV treatment (Figure 2), we tested the hypothesis that *Pg* OMVs disrupt trophoblast–endothelial crosstalk. Initially, we assessed the effect of trophoblast-conditioned media from cells pretreated with *Pg* OMVs and the untreated controls on angiogenesis. The migration of endothelial EA.hy926 cells was quantified using a wound-healing assay. *Pg* OMV-treated trophoblast-conditioned media led to a significant reduction in endothelial cell migration after an 18 h period compared to control trophoblast-conditioned media (Figure 4a). A contributing factor to endothelial dysfunction is its activation and a subsequent enhanced leukocyte adhesion. Therefore, we next evaluated the adhesion of monocytes to endothelial cells as a marker of endothelial activation. As shown in Figure 4b, endothelial cells pretreated with *Pg* OMV Tb CM significantly increased monocyte–endothelial adhesion.

## 4. Discussion

Here, we provide supportive evidence for the role of *P. gingivalis*-derived extracellular vesicles in disrupting the interaction of trophoblast cells with immune and endothelial cells using an in vitro model of the maternal–placental interface. This conclusion is based on the following observations. First, *Pg* OMVs were internalized by first-trimester trophoblast cells and modulated the activity and expression of oxidative and functional markers. Second, trophoblast cells primed with *Pg* OMVs lost their anti-inflammatory effect on neutrophils: An enhanced neutrophil chemoattraction followed by their activation in proinflammatory phenotypes was observed upon incubation with media from *Pg* OMV-pretreated trophoblast cells. Third, reduced migration with enhanced adhesion of monocytes was found in endothelial cells upon incubation with the media from trophoblast cells pretreated with *Pg* OMVs.

A complex regulation of oxidative stress signals was found in trophoblast cells exposed to *Pg* OMVs: High catalase activity and reduced ROS production are both indicative of a protective role of *Pg* OMVs on an excessive oxidative response of the trophoblasts that would impair *Pg* survival. However, there was no effect on GSH levels, and the activity of SOD was not enhanced either, suggesting that transitional oxidative states were induced. This is consistent with the characteristics of this opportunistic anaerobe, which adapts well to the strictly anaerobic environment of the gingival crevice and, at the same time, can set up transition mechanisms of oxygen and peroxide detoxification when leaving its natural environment. These strategies allow it to resist when infecting oxygenated tissue or to face the oxidative burst of neutrophils at the sites of infection [36,37]. Moreover, *Pg* infection altered translation during oxidative stress-induced activation of oral epithelial cells, resulting in differential activation and stress granule composition [38]. Of note, this differential response was observed with both *P. gingivalis*–conditioned media and outer membrane vesicles, implicating a secretory factor in the redox-sensitive transcriptional response, as is the case in our results.

Neutrophils have garnered attention in the last few years, appearing to be either deleterious or beneficial for pregnancy depending on their functional profile [21,34,39,40]. We have demonstrated in previous works that trophoblast soluble factors deactivate neutrophils via inhibition of ROS release and neutrophil extracellular trap (NET) formation [21]. In the present work, we found that soluble factors from *Pg* OMV-primed trophoblast cells further promoted the chemoattraction of neutrophils and, in contrast to their anti-inflammatory effect on neutrophil activation, these media enhanced ROS production paralleled by a higher expression of the membrane subunit of NADPH oxidase gp91^phox^ and the cytosolic subunits p47^phox^ and p67^phox^. A connection between the regulation of NADPH oxidase activity, metabolic pathways during neutrophil activation, and the amounts of ROS released has been reported [35]. Nevertheless, this link within the context of pregnancy and the immunomodulatory role of the trophoblast still requires further investigation.

Finally, a disruptive effect of *Pg* OMVs on the expression of pro-/anti-angiogenic markers in trophoblast cells was paralleled by the inability of trophoblast cells to modulate endothelial function. Certainly, after the priming of trophoblast cells with *Pg* OMVs, trophoblast soluble factors reduced endothelial cell migration and promoted the adhesion of monocytes, reflecting their activation state. The vast transformation of maternal vessels critical for pregnancy outcome occurs under the strict control of trophoblast cells, and failures in this process underly severe adverse pregnancy outcomes like preeclampsia [16]. Much effort has been conducted to identify potential disruptive mechanisms and biomarkers. Among them, the balance of pro-/anti-angiogenic molecules has become a useful tool [32].

Taken together, the effects of *Pg* OMVs shown on the phenotype and function of trophoblast cells along with their impaired interaction with neutrophils and endothelial cells are in line with the proposed deleterious effect of Pg on placentation [13,14,15]. Moreover, the fact that the effects are elicited by OMVs from *Pg* provides new clues to understand the mechanisms operating on trophoblast cells upon Pg infection in early pregnancy since the presence of *Pg* is scarce in the placenta [41]. A recent report showing that OMVs from anaerobic bacteria effectively reach the placenta in normal pregnancies has highlighted the relevance of OMV-mediated effects in healthy and pathologic pregnancies [24]. In the light of this observation and our present results, it is conceivable that the deleterious effects of *Pg* during placentation in periodontitis-associated pregnancies is related to the release of OMVs and their promoting role for subsequent *Pg* infection or disruption of trophoblast cell function.

Regarding the mechanisms involved, it is noteworthy that oxidative stress is modulated in trophoblast cells without affecting cell viability, along with active transcription of specific markers by *Pg* OMVs. The levels of these markers are modulated according to their subsequent effect on the neutrophils’ chemoattraction (MCP-1) and endothelial cell migration (sflt-1 and PlGF). These observations suggest a direct involvement of *Pg* OMVs in the physiology of extravillous trophoblast cells that impacts in their ability to modulate neighboring cells’ profile and function in early pregnancy. Interestingly, the study by Menon et al. [24] referred to above offers new hypotheses of the role of bacterial extracellular vesicles, as it demonstrates their presence in human term normal placenta as well as their incorporation into the placental cells. They even provide evidence on OMV concentration dependency to promote proinflammatory responses in vitro, highlighting the importance of considering not only the presence but also the quantity, cargo, and species involved in the potential effect on pregnancy outcomes. Ongoing studies are designed to dive deeper into these mechanistic hypotheses and perspectives by comparing the effects of OMVs from various periodontal bacteria or non-related strains such as E. coli. In parallel, studies focusing on the major bacterial components or cargo molecules in the vesicles and their downstream effects will add valuable insights into the specificity of *Pg* OMV effects on trophoblast cells and their potential contribution to adverse pregnancy outcomes.

Among the limitations of our study, it is fair to note the use of a human trophoblast cell line to model in vitro the trophoblast–endothelial and trophoblast–immune cell interactions characteristic of early pregnancy. This design allows for in vitro assays using techniques that would be tough to conduct ex vivo in human first-trimester placental samples, on the one hand, and on the other hand, it takes advantage of the fact that the HTR-8 cell line retains key characteristics of first-trimester extravillous trophoblast cells, such as their capacity to invade the decidual tissue and communicate with neighboring cells. Indeed, we have recently shown that *Pg* OMVs reduce the migration and invasion of HTR-8 cells (manuscript accepted, J Cell Physiol. August 2023), supporting the hypotheses tested here that *Pg* OMV-induced changes in cell physiology modify their interaction with vascular and immune cells.

## 5. Conclusions

The effects of *Pg* OMVs on the phenotype and function of trophoblast cells, along with the loss of the anti-inflammatory action on neutrophils and the reduced migration of endothelial cells, are in line with the reported deleterious effect of Pg on placentation. The in vitro model of placentation used allows for the study of potential mechanisms involved in *Pg* infection of the placenta in early pregnancy and contributes to the design of future in vivo and ex vivo approaches.

## Figures and Tables

**Table 1:** Primer sequences. VEGF-a (vascular endothelial growth factor A), PLGF (placental growth factor), IL-6 (interleukin 6), IL-8 (interleukin 8), sFLT-1 (circulating soluble fms-like tyrosine kinase-1), MCP-1 (monocyte chemoattractant protein-1), TNF-α (tumor necrosis factor-α), GP91^phox^ (91-kDa glycoprotein component of the phagocyte NADPH oxidase), P47^phox^ (47-kDa protein component of the NADPH oxidase), P67^phox^ (67-kDa protein component of the NAPDH oxidase), YHWAZ (tyrosine 3-monooxygenase/tryptophan 5-monooxygenase activation protein zeta).

Gene	Sequence	
VEGF-a	Sense	CGCAGCTACTGCCATCCAAT
	Antisense	GTGAGGTTTGATCCGCATAATCT
PLGF	Sense	TGTTCAGCCCATCCTGTGTCTC
	Antisense	CCCAGAACGAACGGATCTTTAGG
IL-6	Sense	TTCGGTACATCCTCGACGGC
	Antisense	TCACCAGGCAAGTCTCCTCA
IL-8	Sense	CCAACACAGAAATTATTGTAAAGC
	Antisense	CACTGGCATCTTCACTGATTC
sFLT-1	Sense	AGAGGTGAGCACTGCAACAA
	Antisense	TCTCCTCCGAGCCTGAAAGT
MCP-1	Sense	CAGCAGCAAGTGTCCCAAAG
	Antisense	GAGTGAGTGTTCAAGTCTTCGG
TNF-α	Sense	GCCTCTTCTCCTTCCTGATCG
	Antisense	CAGCTTGAGGGTTTGCTACA
GP91^phox^	Sense	ACATTCAACCTCTGCCACCAT
	Antisense	ACCCCAGCCAAACCAGAATG
P47^phox^	Sense	CCCACGGACAACCAGACAAA
	Antisense	TCTGACAGAACCACCAACCG
P67^phox^	Sense	CTGTTTGCCTGTGAGGTGTT
	Antisense	AGACACACTCCATCGCCTTG
YHWAZ	Sense	CAGAGAGAAAATTGAGACGGAGC
	Antisense	GTGACTGATCGACAATCCCTTTC

## Data Availability

The data that support the findings of this study can be obtained from the corresponding author upon reasonable inquiry.
